# Drugs As Instruments: Describing and Testing a Behavioral Approach to the Study of Neuroenhancement

**DOI:** 10.3389/fpsyg.2016.01226

**Published:** 2016-08-17

**Authors:** Ralf Brand, Wanja Wolff, Matthias Ziegler

**Affiliations:** ^1^Sport and Exercise Psychology, University of PotsdamPotsdam, Germany; ^2^Department of Sport Science, Sport Psychology, University of KonstanzKonstanz, Germany; ^3^Department of Psychology, Psychological Diagnostics, Humboldt Universität zu BerlinBerlin, Germany

**Keywords:** psychoactive drugs, non-addictive behavior, cognitive enhancement, drug instrumentalization, user types

## Abstract

Neuroenhancement (NE) is the non-medical use of psychoactive substances to produce a subjective enhancement in psychological functioning and experience. So far empirical investigations of individuals' motivation for NE however have been hampered by the lack of theoretical foundation. This study aimed to apply drug instrumentalization theory to user motivation for NE. We argue that NE should be defined and analyzed from a behavioral perspective rather than in terms of the characteristics of substances used for NE. In the empirical study we explored user behavior by analyzing relationships between drug options (use over-the-counter products, prescription drugs, illicit drugs) and postulated drug instrumentalization goals (e.g., improved cognitive performance, counteracting fatigue, improved social interaction). Questionnaire data from 1438 university students were subjected to exploratory and confirmatory factor analysis to address the question of whether analysis of drug instrumentalization should be based on the assumption that users are aiming to achieve a certain goal and choose their drug accordingly or whether NE behavior is more strongly rooted in a decision to try or use a certain drug option. We used factor mixture modeling to explore whether users could be separated into qualitatively different groups defined by a shared “goal × drug option” configuration. Our results indicate, first, that individuals' decisions about NE are eventually based on personal attitude to drug options (e.g., willingness to use an over-the-counter product but not to abuse prescription drugs) rather than motivated by desire to achieve a specific goal (e.g., fighting tiredness) for which different drug options might be tried. Second, data analyses suggested two qualitatively different classes of users. Both predominantly used over-the-counter products, but “neuroenhancers” might be characterized by a higher propensity to instrumentalize over-the-counter products for virtually all investigated goals whereas “fatigue-fighters” might be inclined to use over-the-counter products exclusively to fight fatigue. We believe that psychological investigations like these are essential, especially for designing programs to prevent risky behavior.

## Introduction

Use of psychoactive drugs is common in most societies. Use of caffeine, alcohol, and nicotine is particularly widespread; illicit drugs such as cocaine or marijuana are consumed less frequently (Kandel et al., 1997). There is disproportionate growth in medically unsupervised use (i.e., abuse) of prescription drugs, particularly opioids and stimulants, especially among adolescents and young adults (Johnston et al., 2010; United Nations, 2011).

Psychological research on motivations for using psychoactive drugs is often concerned with addiction and theories of drug use often focus on addiction (e.g., O'Brien et al., 1992; Koob and LeMoal, 1997; Baker et al., 2004). Given the known costs of addiction, both for the individual and for society, it is clearly an important research target. Many drug users should not be considered addicted however; for example 95% of alcohol consumers (Anderson and Baumberg, 2006), around 92% of nicotine users (Baumeister et al., 2008) and 91% of caffeine users (Meredith et al., 2013) should not be considered addicted^1^. It is likely that similar figures apply to abuse of prescription drugs (United Nations, 2011).

The starting point for our investigation was the growing number of research articles on university students' use of psychoactive pharmacological products for the purpose of enhancing cognitive performance. It has been reported that 6-8% of university students in Germany (Middendorff et al., 2015) and perhaps the same or an even higher proportion in the United States (Smith and Farah, 2011, report a rather uninformative guestimate of 2–50%) have abused drugs such as Modafinil (a wakefulness-promoting drug usually prescribed to treat shift-work sleep disorder and narcolepsy) for this purpose. Recently the presumed motivation for such drug use has prompted research on the cognitive effects of pharmaceutical drugs (e.g., benchmarking effect sizes of different dopaminergics; Fond et al., 2015) as well as several nutraceuticals (e.g., Ginseng and Bacopa benchmarked against Modafinil; Neale et al., 2013) and the ethics of usage (e.g., whether safe pharmacological enhancement could help resolve societal inequalities; Glannon, 2015). Research on students' motivation to try and perhaps subsequently persist with using such performance enhancing substances is much less elaborated. This research aimed to investigate substance users' motivated behavior systematically, i.e., from a psychological perspective.

### Drug instrumentalization theory

Drug instrumentalization theory (DI theory; Müller and Schumann, 2011a,b) suggests that non-addictive drug use can be explained in functional terms, as a purposeful, goal-directed process. For example the wakefulness-promoting prescription drug Modafinil might be used to enhance academic performance. It is a matter of fact, however, that some students prefer to use caffeinated, non-prescription products for this purpose (Franke et al., 2011). Others might know that a strong cup of filter coffee (Walsh et al., 1990) is at least as effective a stimulant as many caffeinated over-the-counter products and perhaps more so, and prefer this option. DI theory suggests that the starting point for explaining the non-addictive use of drugs should be to consider the purpose for which they are taken; before considering the specific characteristics of the various substances that could be used for that purpose. DI theory proposes a non-exhaustive list of goals relevant to instrumental drug use; these goals are presented in Table 1 along with examples from the domains discussed here and in the following sections.

**Table 1:** **Instrumentalization goals as proposed by DI theory (Müller and Schumann, 2011a,b) with behavioral examples**.

**No**.	**Instrumentalization goal**	**Label^a^**	**Behavioral example^b^**
[1]	Improved cognitive performance	Cognitive performance	Using methylphenidate to feel more concentrated and alert
[2]	Counteracting fatigue	Fatigue	Using caffeine to counteract fatigue
[3]	Improved social interaction	Social interaction	Using alcohol or other drugs at parties to be more talkative, disinhibited, and self-confident
[4]	Facilitated sexual behavior	Sexual behavior	Using drugs like alcohol or cocaine to increase the likelihood of and pleasure during sexual intercourse
[5]	Facilitated recovery from psychological stress	Stress recovery	Using cannabis to recover from a stressful day at work
[6]	Coping with psychological stress	Stress coping	Using alcohol to reduce perceived stress level before an important meeting
[7]	Euphoria and hedonia	Euphoria	Using cannabis, alcohol, or other to induce intense well-being and positive feelings
[8]	Self-medication for mental problems	Self-medication	Using antidepressants, cannabis or alcohol to reduce depressive symptoms, regain control over one's mental state, and enhance functioning in everyday life
[9]	Sensory curiosity and facilitating spiritual and religious activities	Sensory curiosity	Using hallucinogenic drugs (e.g., MDMA) to facilitate spiritual experiences

a *Short labels for the goal detailed in the previous column*.

b *This list of examples is illustrative rather than exhaustive*.

Another claim of DI theory is that repeated, non-addictive drug use should be modeled as a two-step process: “(1) the seeking and consumption of a psychoactive drug in order to change the present mental state into a previously learned mental state, which then allows for (2) better performance of other, previously established behaviors and better goal achievement” (Müller and Schumann, 2011a, p. 295). Whilst we largely endorse the proposed first step, we think that from a psychological perspective the second step needs readjustment with regard to the qualifier “better” that implies factual improvement in performance and goal achievement.

Subjective expectations are important determinants of human behavior (e.g., Armitage and Conner, 2001). We argue that the presumed functions of a substance are an essential factor in motivation and perhaps even more important than the chosen substance's subsequent effects on performance (Wolff and Brand, 2013). This behavioral approach (Wolff and Brand, 2013; Wolff et al., 2014; Brand and Koch, 2016) differs from more substance-based approaches adopted by other authors (e.g., Franke et al., 2014; Maier and Schaub, 2015). It is our view that—in the terminology of learning theory—drug use is reinforced by the *subjective state* that this behavior, which was intended as a means to an end, produces. This reinforcement is moderated by the physiological and other observable effects of the drug which thus influence subsequent usage; a drug which proves more effective in producing the desired goal might come to be used more frequently.

This account implies, however, that objectively “better” performance and goal achievement is not a necessary consequence of instrumental drug use. We therefore suggest modifying the proposed claim about how individuals instrumentalize drugs to: (1) the seeking and consumption of a potentially psychoactive drug with the aim of reinstating a previously learned mental state that allows for (2) subjectively enhanced goal achievement.

### Instrumental use of psychoactive drugs to enhance cognitive performance: one aspect of neuroenhancement

One aim of this article is to embed the active debate on what has been called pharmacological “cognitive enhancement” (e.g., Hildt and Franke, 2013) in the broader context of DI theory's (Müller and Schumann, 2011a,b) framework theory for non-addictive psychoactive drug consumption.

Enhancements can be tried with the aim of enhancing cognitive functioning (e.g., working memory, task flexibility) and enabling increased effort (e.g., in order to stay awake and study longer; see Zelli et al., 2015), aims which might be regarded as analogous to two of the DI theory instrumentalization goals, “improved cognitive performance” and “counteracting fatigue.” One might try to attain these goals by using a suitable over-the-counter product, e.g., caffeine pills, herbal substances; however some people regard over-the-counter medication as being fine for mild or occasional symptoms but less suited to treatment of severe symptoms, for which more potent drugs are necessary (United Nations, 2011). These individuals might also believe that recognized medical drugs are safer than illicit drugs, even when used unsupervised and hence although they would be unwilling to try the illegal drug “speed” (amphetamine), they might decide to try Modafinil (a prescription drug) in an attempt to enhance cognitive performance or counteract fatigue.

It is obvious from this example that diverse substances can be used in pursuit of the same goal (equifinality). It is also possible to use a single drug as an instrument for attaining several different goals (multifinality); for example cocaine users report using this illicit substance to enhance cognitive performance, as well as to facilitate social interactions and induce euphoria (Boys et al., 2001). Research focused on the use or abuse of pharmacological products to enhance cognitive performance has so far largely neglected this second aspect, multifinality, of instrumental drug use (e.g., Mazanov et al., 2013; Franke et al., 2014; Sattler et al., 2014; Wolff et al., 2014).

This point, the widely neglected aspect of multifinality in the respective studies, calls into question current usage of the terms “cognitive enhancement.” It unjustifiably narrows the phenomenon under investigation. Similar criticisms have been made by researchers who note that many of the substances used for “cognitive enhancement” are not very effective for this purpose (Zohny, 2015). We propose using the umbrella term *neuroenhancement* (*NE*) instead^2^. It is important to emphasize our suggestion that using this term in the proposed way thus refers to a *behavior* that is *explicitly connected* with a *specific goal*: We define this behavior, NE, as the non-medical use of psychoactive substances (and technology; e.g., Clark and Parasuraman, 2014) for the purpose of producing a subjective enhancement in psychological functioning and experience.

It is important to note that in pursuit of, for example, enhanced cognitive performance, individuals may instrumentalize any substance or technology which they think might help them to reach their goal. The attribution of relevant efficacy to the substance or technology is sufficient to qualify their behavior as attempted NE behavior and to investigate this behavior's motivational roots (Wolff and Brand, 2013).

### This study

Building upon the above-described argument, in the first stage of our empirical study we explored user behavior by analyzing patterns of relationships between chosen drug options (“over-the-counter products,” “prescription drugs,” and “illicit drugs”; e.g., Franke et al., 2014) and instrumental goals (“better cognitive performance and reduced fatigue,” “better social interaction,” “facilitation of sexual behavior,” “enhanced recovery from and coping with psychological stress,” “euphoria and hedonia,” “more attractive physical appearance,” “self-medication for mental problems,” “sensory curiosity and facilitation of spiritual and religious activities”; Müller and Schumann, 2011a). We aimed to find and then confirm empirical patterns that would help us to address the question of whether NE behavior should be considered a goal-directed behavior in which the choice of drug is predicated on its presumed functionality in relation to that goal or whether the choice of a drug option (e.g., an over-the-counter product but not an illicit drug) is primarily driven by other factors.

The second stage of our analysis explored whether users could be segregated into qualitatively different groups on the basis of the combination of the psychological variable “goal” and the attribute “drug option” (they were classified in these terms in the first stage). We did this because inter-individual differences are important when it comes to monitoring and preventing risky behaviors (cf. Kreuter and Wray, 2003; Rimer and Kreuter, 2006).

In summary, we hoped to make a theoretically informed contribution to the psychological literature which would help to define the boundaries of NE research and provide empirical evidence which could be used to inform programs targeting the misuse of problematic substances (e.g., Wilens et al., 2008).

## Methods

### Study sample

The focus here was on university students. A non-exhaustive manual search of the internet resources of public and private German, Swiss and Austrian universities yielded the email addresses of 853 student associations for study programs in Biology, Computer Science, Economics, Educational Sciences, English and German language and literature studies, Electrical Engineering, Health Sciences, Law, Mathematics, Medical Sciences, Physics and Psychology. These student associations were contacted and asked to distribute the link to our online questionnaire using their student mailing lists. We are unable to assess how many student associations from which universities actually complied with this request.

Participation was voluntary and no compensation was offered for participation. Participants were informed that they would be able to complete the questionnaire anonymously (i.e., without giving their name or contact address). They were also informed in advance that they could decide to stop working through the questionnaire at any time without disadvantaging themselves in any way and that their answers would not be stored unless they clicked the “send data” button at the end of the questionnaire. The study was carried out in accordance with recommendations of the ethical committee of the University of Potsdam.

In total, 2771 students began working through the questionnaire. Around 50% (*n* = 1438) completed it and sent us their answers. The mean age of this group of responders was 23.95 ± 5.43 years; 950 (66%) were women. We did not collect data on the study programs in which these participants were enrolled.

### Measures

Drug instrumentalization was assessed separately for each goal. Participants were first asked if they had ever used any substance to achieve a given goal. Participants then responded to three dichotomous (yes/no) items relating to whether they had already used an over-the-counter product, a prescription drug or an illicit drug in pursuit of this goal. We decided to assess the goal “enhancement or rebuilding of cognitive performance” with two questions (one for the “enhancement of…” and one for the “rebuilding of…” aspect) as these statements reflect distinct processes; the goal “facilitated recovery from and coping with psychological stress” was treated similarly. Participants thus indicated their pattern of behavior with respect to 27 goal × drug option combinations. In the remainder of the article we will refer to this set of items as the DI questionnaire.

### Statistical analyses

All statistical analyses were conducted using the statistical programs R (R Development Core Team, 2013) and MPlus 7 (Muthén and Muthén, 2013). The factorial structure of drug instrumentalization as assessed by the DI questionnaire (instrumentalization goals × drug option) was explored using exploratory factor analysis (EFA; psych package; Revelle, 2014) and confirmed using confirmatory factor analysis (CFA; lavaan package, Rossel, 2012). The dataset was randomly split in half to allow for independent EFA and CFA. Model tests were done according to the guidelines of Beauducel and Wittmann (2005), Hu and Bentler (1999), and Heene et al. (2011). We looked at the global model test as well as the fit indices *RMSEA* (< 0.05), *SRMR* (< 0.08), and *CFI* (= 0.95). A robust *ML* estimator was used to correct for violations of multivariate normal distribution. Missing data were dealt with using the FIML method. After this the complete dataset was subjected to factor mixture model (FMM) analysis to determine whether the latent structure was person-homogeneous, in other words to find qualitatively different groups of users. FMMs have several advantages over traditional methods of latent class identification. Specifically, FMMs allow drug instrumentalization to be modeled as an individual difference variable within a CFA model (e.g., Leite and Cooper, 2010). FMMs can be used for the analysis of data with underlying continuous constructs whilst simultaneously modeling population heterogeneity as they incorporate categorical and continuous latent variables (Lubke and Muthen, 2005, 2007). The procedure we followed in calculating and reporting our FMM analysis has been described in more detail elsewhere (Ziegler et al., 2015).

## Results

### Descriptive statistics

**Figure 1:**
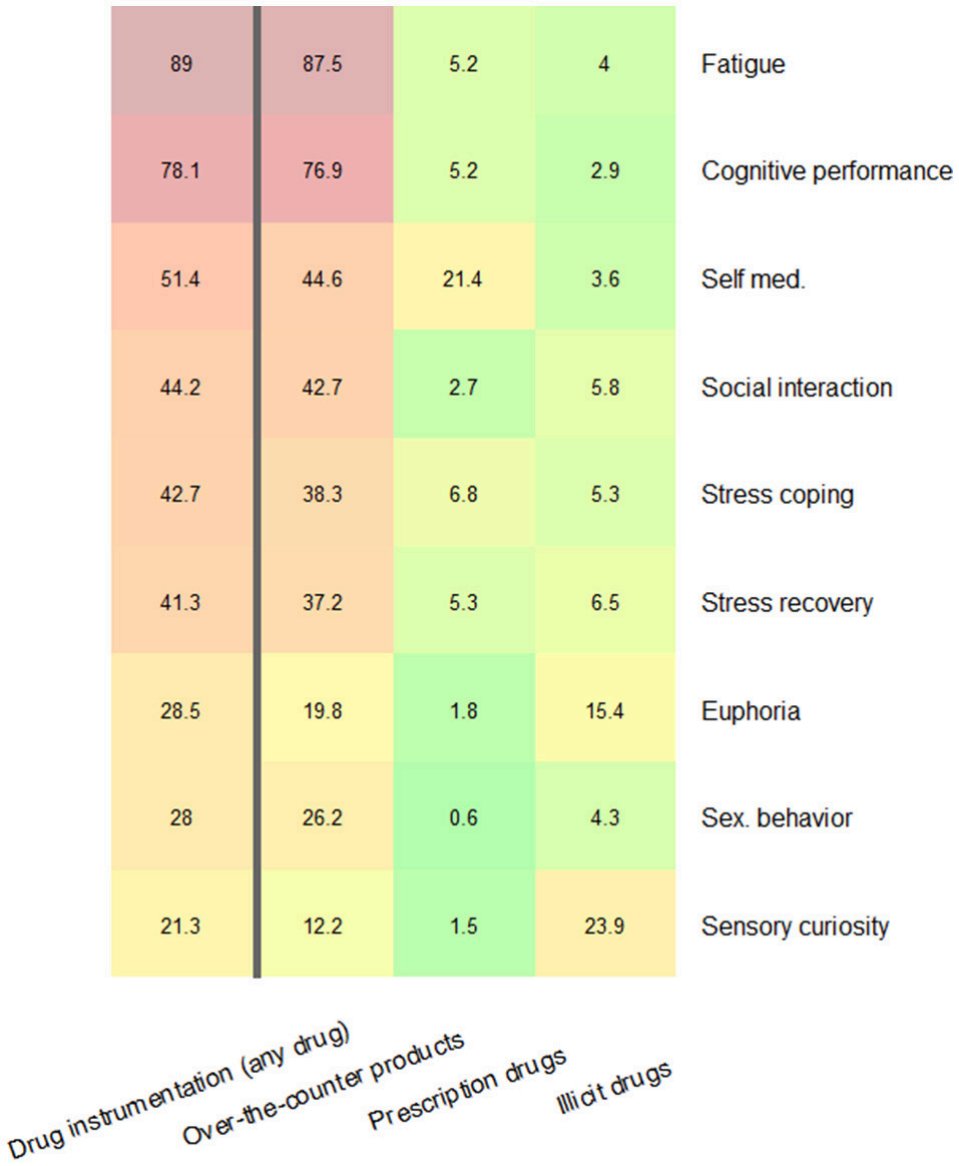
**Prevalence of instrumental use of drugs in pursuit of each goal irrespective of drug option (left column) and as a function of the three drug options (three right columns)**. Multiple positive responses were possible and therefore values in the colored columns do not add up to the values presented in the left column.

Descriptive statistics for all investigated variants of drug instrumentalization are visualized as a heat map in Figure 1. First, descriptive statistics indicated that all investigated goals were instrumentalized by at least some of the sample (the goal instrumentalized by the smallest proportion was “improving physical appearance”: 18.6%). Second, answers indicated that in this sample all three drug options were employed in pursuit of the goals we investigated. Third, there was large variation between the frequencies with which specific “goal × drug option” configurations appeared; for example 87.2% of our participants reported that they had used over-the-counter products to fight fatigue but only 0.6% reported that they had used prescription drugs to facilitate sexual encounters.

### Factor analyses

**Figure 2:**
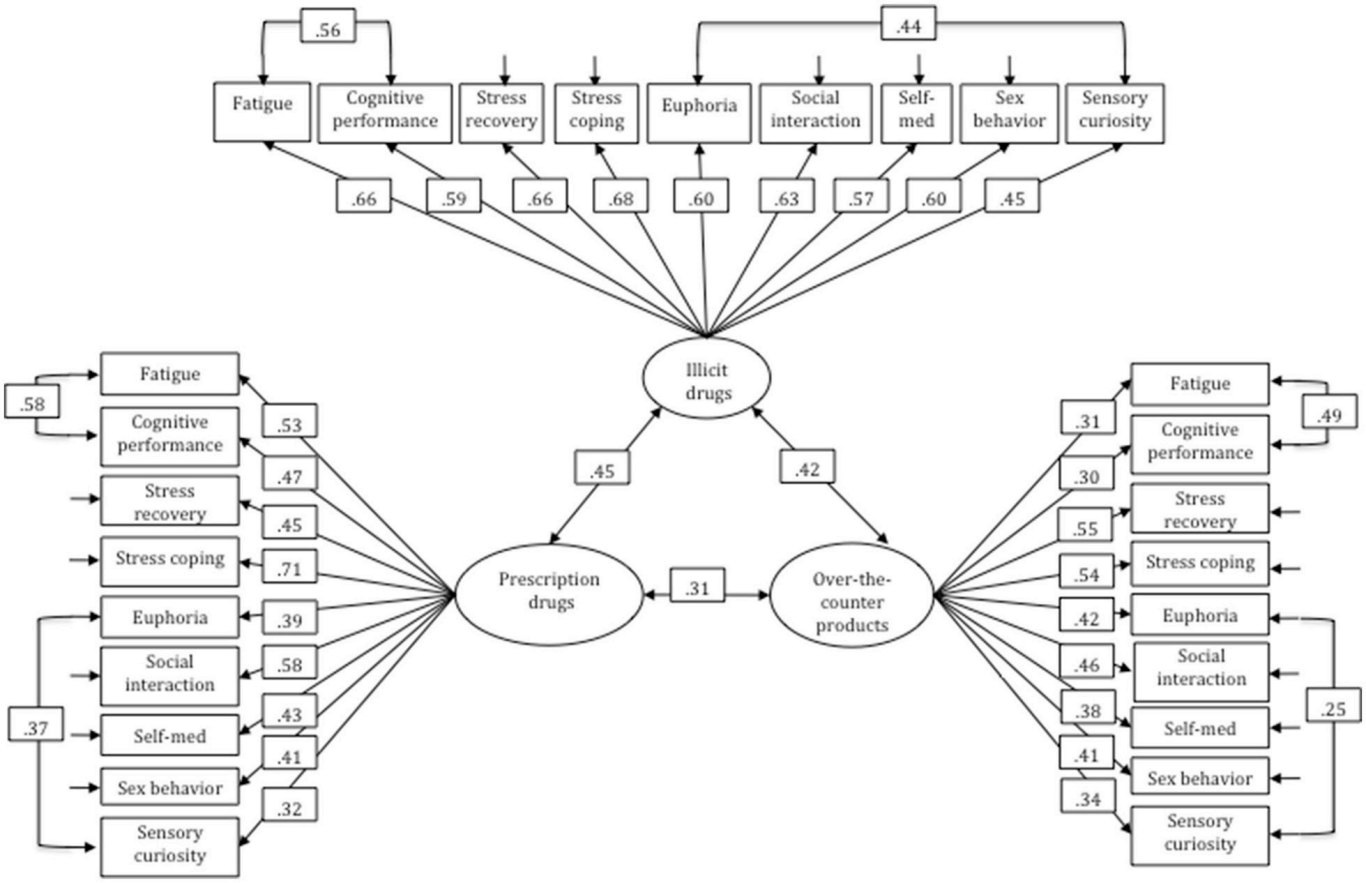
**The full three-factor model for DI behavior based on the CFA**.

Parallel analysis (Horn, 1965) suggested that seven factors could be extracted. The minimum average partial test (Velicer, 1976) suggested a three factor solution. To choose a solution the two- to seven-factor solutions were extracted using principal axis factoring and geominT rotation with the R package psych (Revelle, 2014). The three-factor solution was the most plausible, reflecting patterns held together by the three drug options “over-the-counter products,” “prescription drugs,” and “illicit drugs.” Factor loadings for this solution are given in Table 2. Consequently, in the subsequent CFA we tested this model, labeling the three factors “over-the-counter DI,” “prescription DI,” and “illicit DI.” In the first step we tested the three measurement models for each factor separately, following advice by Ziegler and Hagemann (2015) according to which misfit within single measurement models might be harder to detect in the complete model otherwise. In each factor measurement model the nine items relating to whether a given drug option had been used to achieve specific goals were included in the analyses. The items and loadings for each factor measurement model are shown in Table 3. Analyses of model fit indicated that in all three cases measurement models described the data well (Table 3). We then added the same correlated residuals to all the measurement models (“fatigue” with “cognitive performance”; “euphoria” with “sensory curiosity”) and ran a final analysis to assess the fit of the overall model (Figure 2). In order to achieve acceptable model fit three correlated error terms had to be included (“self-medication using prescription drugs” with “self-medication using over-the-counter drugs”; “sensory curiosity using illicit drugs” with both “sensory curiosity using of over-the-counter drugs” and “sensory curiosity using prescription drugs”). The fit indices for the complete model were χ^2^ = 759.44, *df* = 312, *p* < 0.001; *CFI* = 0.90; *RMSEA* = 0.03; *SRMR* = 0.06. This analysis indicated that the 27-item DI questionnaire was best be described by three drug option factors, each consisting of nine items with an identical format reflecting nine different aspects of drug instrumentalization and hence that drug instrumentalization behavior is primarily accounted for by the drug option rather than by specific instrumental goals.

**Table 2:** **The exploratory three-factor model for responses to the DI questionnaire**.

**Drug-types × DI goals**	**Factor 1**	**Factor 2**	**Factor 3**	**h^2^**	**u^2^**
**ILLICIT DRUGS**					
… × Fatigue	0.70	0.24	−0.06	0.54	0.46
… × Stress coping	0.57	0.03	0.08	0.33	0.67
… × Stress recovery	0.60	0.08	−0.01	0.36	0.64
… × Cognitive performance	0.61	0.24	−0.03	0.43	0.57
… × Euphoria	0.65	−0.03	0.16	0.45	0.55
… × Sex. behavior	0.47	0.17	0.04	0.25	0.75
… × Self-med	0.44	0.02	0.08	0.20	0.80
… × Social interaction	0.54	0.07	0.16	0.32	0.68
… × Sensory curiosity	0.54	0.02	0.19	0.33	0.67
**PRESCRIPTION DRUGS**					
… × Euphoria	0.31	0.16	0.09	0.13	0.87
… × Sensory curiosity	0.38	0.04	0.15	0.17	0.83
… × Stress coping	0.09	0.80	0.07	0.66	0.34
… × Fatigue	0.24	0.61	−0.02	0.43	0.57
… × Cognitive performance	0.28	0.60	0.00	0.44	0.56
… × Social interaction	0.13	0.53	0.00	0.30	0.70
… × Stress recovery	0.05	0.45	0.13	0.22	0.78
… × Self-med.	0.08	0.38	0.14	0.17	0.83
… × Sex. behavior	0.24	0.31	0.02	0.15	0.85
**OVER-THE-COUNTER SUBSTANCES**					
… × Sensory curiosity	0.34	−0.04	0.33	0.22	0.78
… × Euphoria	0.24	−0.03	0.37	0.19	0.81
… × Stress coping	0.12	0.11	0.47	0.24	0.76
… × Stress recovery	0.14	0.11	0.39	0.19	0.81
… × Social interaction	0.21	0.07	0.38	0.19	0.81
… × Self-med.	−0.03	0.08	0.37	0.14	0.86
… × Sex. behavior	0.20	0.01	0.30	0.13	0.87
… × Fatigue	0.03	−0.05	0.46	0.21	0.79
… × Cognitive performance	0.03	0.00	0.47	0.22	0.78

**Table 3:** **Factor loadings for three CFA measurement models and fit indices for these models**.

	**Latent factor**		
	**Over-the-counter substances**	**Prescription drugs**	**Illicit drugs**
**STANDARDIZED FACTOR LOADINGS**			
Fatigue	0.31^*^	0.48^*^	0.65^*^
Cognitive performance	0.30^*^	0.45^*^	0.62^*^
Stress recovery	0.57^*^	0.44^*^	0.62^*^
Stress coping	0.53^*^	0.68^*^	0.68^*^
Euphoria	0.46^*^	0.46^*^	0.61^*^
Social interaction	0.51^*^	0.62^*^	0.59^*^
Self-med.	0.39^*^	0.43^*^	0.55^*^
Sex. behavior	0.41^*^	0.39^*^	0.52^*^
Sensory curiosity	0.36^*^	0.35^*^	0.46^*^
**FIT INDICES**			
χ^2^ (*df*)	80.44^*^ (25)	166.22^*^ (25)	68.33^*^ (25)
CFI	0.93	0.92	0.98
RMSEA	0.056	0.087	0.05
SRMR	0.041	0.047	0.028

* *p < 0.05*.

### Factor mixture models

Building on differentiation of our three latent factors of drug instrumentalization, the second goal was to investigate whether latent variables differentiating between types of instrumental drug users could be identified. Simply put, we were interested in whether qualitatively different classes of functional drug use could be identified with respect to each of the three drug options. Separate FMMs consisting of the nine items relating to use of each drug option (over-the-counter products; prescription drugs, illicit drugs) for DI were tested. Loadings on the latent usage variable were assumed to be equal for all classes in order to ensure that a similar latent variable was measured (factorial invariance). A robust maximum likelihood estimator was used to address the non-normality of the data. As to the acceptable model fits of the measurement models underlying these analyses (see Table 3) problems due to the exploitation of residual patterns are unlikely (Bauer and Curran, 2004). There was marginal evidence for the validity of a two-class solution, and only in the case of the over-the-counter DI factor (Lo-Mendel-Rubin test: *p* = 0.058; adjusted Lo-Mendel-Rubin test: *p* = 0.059)^3^. This suggests that we have two qualitatively different classes of users within the latent factor of over-the-counter products in our data. The values of the intercepts revealed that the average responses of the classes were different for almost all items. The first class (87.5% of the participants in our sample) could be described as having a higher propensity to use over-the-counter products in pursuit of diverse goals (Table 4); we termed this class of users “neuroenhancers.” The second class of users had a generally lower propensity to use over-the-counter products (as indicated by the much lower intercept values in Table 4) and used over-the-counter products almost exclusively to fight fatigue; this class of users was termed “fatigue-fighters.”

**Table 4:** **Descriptive statistics for class solutions of the factor mixture models**.

	**Class I**		**Class II**	
**DI-Goals**	Intercept^*^	**S.E**	Intercept^*^	**S.E**
Social interaction	0.432	0.014	0.350	0.050
Sex. behavior	0.269	0.013	0.183	0.042
Cognitive performance	0.879	0.009	−0.017	0.020
Fatigue	0.854	0.010	0.981	0.022
Stress coping	0.408	0.014	0.161	0.052
Stress recovery	0.383	0.014	0.244	0.059
Self-med.	0.457	0.014	0.333	0.052
Sensory curiosity	0.123	0.009	0.092	0.026
Euphoria	0.193	0.011	0.193	0.011

* *p < 0.001*.

## Discussion

The aim of this study was to develop a theoretical conception of NE behavior that would account for original empirical data on drug instrumentalization among university students. The patterns of participants' responses to the DI questionnaire suggested that NE behavior is probably based on a primary decision about usage of a class of drugs (drug option).

In other words the EFA and CFA suggested that rather than identifying a goal or motivation (e.g., “I want to fight tiredness”) and then instrumentally using the different drug options that might enable them to achieve this goal (e.g., to identify the most effective one) individuals seem to instrumentally use a given drug option and then accept the constraints this places on goal attainment (e.g., “I am willing to use over-the-counter products but not to abuse prescription drugs even if this limits how effectively I can fight my tiredness”).

Results from the FMM analysis can tentatively be interpreted as supporting the notion of two qualitatively different classes of users. Both of them predominantly used over-the-counter products; they were termed “neuroenhancers” and “fatigue-fighters.” “Neuroenhancers” were characterized by a higher propensity to instrumentally use over-the-counter products for virtually all the goals specified in DI theory (improving cognitive performance and overcoming fatigue were endorsed with the largest propensity; Table 4). In contrast “fatigue-fighters” seemed to instrumentalize over-the-counter products solely for fighting fatigue. No comparable qualitative difference in patterns of usage was found among users of prescription drugs and illicit drugs.

DI theory provided the framework for this research. We started by asking participants about their instrumental use of over-the-counter products and their abuse of prescription and illicit drugs for the goals specified in DI theory. We did not ask about any other kind of drug use. In our sample of university students we found evidence that in the group of participants all drug options were used for all the proposed goals. In our view this finding corroborates one of the central claims of DI theory, namely that individuals' instrumental use of drugs cannot be adequately explained—or investigated—without addressing the specific goal(s) which motivated this use. Although users might respond positively when asked if they have used a given drug to enhance their cognitive performance, other co-existent goals might better account for their behavior. Studies of people's motivations or reasons for using drugs that they believe have the potential to enhance cognitive performance should therefore not be limited to consideration of this particular goal. This study revealed that *multifinality*, i.e., using one instrument to pursue several goals, is an important pattern of behavior in the context of use of psychoactive substances to produce a subjective enhancement in psychological functioning and experience, i.e., neuroenhancement.

### Discussion of factor analyses results

Factor analyses revealed the existence of three drug option-related factors, over-the-counter product DI, prescription drug DI, and illicit drug DI, but no goal-related factors. The various instrumentalization goals appeared in each of the three drug option factors instead. This indicates that participants' primary decision related to the drug option(s) they were willing to use instrumentally. In practice this meant that if, for example, an individual resorted to using an over-the-counter product in an attempt to enhance cognitive performance then he or she was more likely to use over-the-counter products in pursuit of some other goal. An alternative pattern of results would have been that the primary decision was about which goal to pursue via use of drugs and secondarily what drug option might be the most effective tool for achieving that goal. Such a pattern would have been reflected in a set of factors representing different instrumentalization goals (or patterns of goals). A third possibility is that there might have been systematic links between drug options and specific goals, e.g., the use of over-the-counter products for facilitation of social interaction and using prescription drugs for facilitating sexual encounters. We did not observe this kind of goal-dependent switching between drug options in our sample. Our preliminary, cautious interpretation of these results, in terms of instrumental (i.e., means-end) drug use, is that individuals use drugs as instruments for pursuing a variety of goals, but that willingness to instrumentalize a drug option takes priority over attainment of a specific goal in the decision-making process. Although we found marked differences in the frequency with which specific drug options were chosen as tools for pursuing specific goals on a descriptive level, factor analyses revealed that there was more consistency in the type of instrument an individual chose, irrespective of goal. It is possible that individuals' attributions of functionality are general to a drug option and aligned with their usage behavior, for example, an individual who believes that only prescription drugs are both powerful and safe enough to allow to enable one to attain one's objectives might use methylphenidate (instead of a simple energy drink) to enhance his or her concentration and would similarly choose to use prescription antidepressants (rather than *Ginkgo biloba* products) to enhance his or her subjective quality of life.

Generally speaking, one result is that the observed variance-covariance matrix was best explained by three correlated factors representing the three different drug options. The more inclined an individual is to use a given drug option for one specific goal, the more likely it is that he or she will choose the same option as an aid to attaining other goals. In contrast, a willingness to use one option as an instrument for attaining a specific goal, e.g., an over-the-counter product to facilitate sexual behavior, does not imply a similar willingness to use other options, e.g., illicit drugs, in pursuit of that goal.

### Discussion of factor mixture modeling results

We found some support for the idea of two different user classes for over-the-counter products. The two classes could be described in terms of “neuroenhancers” and “fatigue-fighters.” The possible existence of two qualitatively different classes of user indicates that individuals differ not only with respect to what options they are willing to use for DI—as the factor analyses showed—but also, in the case of use of over-the-counter products, with respect to what goals they pursue using drugs. The class of participants who were inclined to use drugs in pursuit of a variety of goals (the neuroenhancers) seems to see drugs as effective instruments for pursuing the rather general goal “modulation of performance.” The second class seems to consist of individuals who only use drugs as instruments for “staying awake” (the fatigue-fighters) and largely abstain from other forms of instrumental drug use.

We suggest—although at this stage it is only a hypothesis—that “neuroenhancers” use drugs proactively, and truly as enhancers i.e., in pursuit of supra-normal performance, whereas “fatigue-fighters” use drugs more reactively, as a means of overcoming a deficit (sub-normal performance). In future research it will be interesting replicate the two class solution we observed and to investigate the drivers behind (these) different patterns of behavior.

Latent classes were only identified within the over-the-counter product DI factor. At present we can only speculate about why no latent classes were identified within the other DI factors. One possible reason is that instrumental use of prescription and illicit drugs is a more socially sensitive behavior than instrumental use of over-the-counter products (Dietz et al., 2013) and thus we failed to detect latent user classes within the other DI factors because participants did not report their use of these drug options truthfully. Similarly, the low rates of use of these drug options might have made it impossible to distinguish different classes of users. In our sample the reported prevalence of drug use in pursuit of the DI goals more directly related to academic performance (counteracting fatigue, enhancing cognitive performance, stress recovery) was comparable with previous reports (cf. McCabe et al., 2005; Mache et al., 2012). In our opinion there is a second plausible explanation for the failure to detect different latent classes of user in the cases of prescription and illicit drugs. Given the generally lower prevalence of DI using prescription drugs and illicit drugs, it is possible that different classes of latent user have simply not yet emerged in society. This might be because the only legally obtainable drugs which are generally known as instruments for attaining the various goals specified in DI theory (regardless of their actual efficacy) are over-the-counter products. In other words the university students in our sample might consider themselves “experts” on DI with over-the-counter products but not with the other drug options. In future research it would be interesting to investigate whether “knowledge about drugs” and “drug availability” emerge as latent classes in analysis of DI.

### Drug instrumentalization in this sample

Our sample was a self-selected convenience sample of university students and therefore does not permit inferences about the general population. Nevertheless, our recruitment strategy targeted students studying the most popular academic subjects in Germany, Switzerland and Austria; we were thus able to recruit a large, diverse sample of university students.

We found empirical support for instrumental use of drugs in pursuit of all the goals specified in DI theory. The reported lifetime prevalence of use of any drug in pursuit of goals varied enormously between goals. The majority of our participants had used drugs as instruments to counteract fatigue (89.0%) and enhance cognitive performance (78.1%). Drug instrumentalization with respect to certain goals seems to be the norm amongst the student population, whereas instrumental use of drugs in pursuit of others is relatively uncommon. One straightforward explanation for these differences is that some goals were of greater personal importance to our sample than others. This might also account for the recent spike in public attention (e.g., Partridge et al., 2011; Rath, 2012) and scientific attention to performance enhancement and its reported prevalence in academia (e.g., Maher, 2008). The two goals most commonly pursued via drugs in our study are very closely linked to the domain of structured learning. The relative frequency of instrumental drug use in pursuit of these goals might simply reflect the heightened importance of academic performance in society.

An alternative explanation is that the observed differences in how frequently goals are pursued via drugs reflect subjective perceptions of what drug options are most effective for which goals. The drug options most frequently used for all the goals we investigated was over-the-counter drugs. The most frequently targeted goals might represent those which folk psychopharmacology connects most closely with over-the-counter products, namely “overcoming fatigue,” “improving cognitive performance,” “coping with stress,” “recovering from demands,” and “facilitating social interaction.” Prescription drugs were used most frequently for “self-medication” and illicit drugs were used most frequently for “sensory curiosity.” There is intuitive appeal to this account, as it implies that individuals choose substances that are generally thought to be effective for the goals in which they are interested. If one wants to self-medicate for mental problems, prescription drugs are the most promising candidate as they are marketed (and designed) as effective treatments for mental problems. Similarly, illicit substances are commonly perceived as a good way of attaining a euphoric state.

A very important issue that needs to be resolved by further investigations however is that moral intuitions, perceptions about cultural tolerance and acceptable risk-taking, together with institutional and societal ambivalence to enhancing substances (and illicit drugs especially) might differ between countries and cultures. The phenomenology we found in our European sample might not correspond with the situation in Arab countries (e.g., Wolff et al., 2016). Cross-cultural comparisons should be conducted to shed light on this.

### Limitations

This study used DI theory as the basis for research into the psychology of drug instrumentalization. We feel our results provide some important insight into the kinds of means-end (i.e., instrumental action-goal) relationship. Some limitations of the research should, however, be discussed along with questions that remain to be addressed in future research.

Goals and drug options might differ in terms of their social desirability and hence the extent to which relevant behavior is over- or under-reported. Randomized response technique (RRT, Greenberg et al., 1969) is a method of maximizing respondent anonymity in order to reduce the impact of social desirability bias on responses. This method was not suitable for our purposes as it is impossible to infer affirmation or denial of a certain behavior on the individual level from this type of data. We could not have investigated the factorial structure of drug instrumentalization or identified latent drug use classes with data collected using RRT. Use of indirect indicators is another option for dealing with social desirability bias (Greenwald et al., 2009), for example, the Implicit Association Test (Greenwald et al., 1998) has been shown to be valid predictor of athletes' doping test results (Brand et al., 2014) that is hard to distort (Wolff et al., 2015). Further studies should investigate whether indirect tests are needed or helpful in acquiring valid self-report data on NE behavior.

We did not ask for information about exactly which drugs university students had used for drug instrumentalization. We were thus not able to make assessments on specific substances. DI behavior seems to be driven largely by an individual's perception of the functions of a drug option rather than by its objective functional profile. This does not imply that future studies should refrain from assessing the use of specific drugs. Information about what drugs are perceived as effective instruments for attaining certain goals would be valuable.

It has been shown that FMM analyses can yield artificial solutions in case of non-normality or when ill fitting models are analyzed (Bauer and Curran, 2004). Even though the models analyzed here had acceptable model fit and a robust maximum likelihood estimator was used, the results should be interpreted with care. The 2-class solution fit better than a 1- or 3-class solution. Still, the direct test of significance was only marginally significant with the given sample size. Moreover, considering our questionnaire format, it cannot be ruled out that minor dependencies between items occured. Considering the explorative nature of this study as well as the high plausibility of its findings, the 2-class solution should be regarded as a feasible working hypothesis at least. Thus, future research should replicate our finding trying different questionnaire formats and more diverse samples.

### Practical implications and conclusion

Knowing what an individual hopes to achieve by using a drug enables one to take a more informed approach to dealing with such behavior; this might involve endorsement, monitoring, preventive strategies, treatment, or prohibition. For example, use of an illicit drug for self-medication might warrant a different response from use of the same drug for hedonistic purposes. Another issue is that ethical evaluation of different DI goals might be perceived ambiguous in parts of the society. For example doping in sport (although not yet explicitly labeled as a DI behavior) is widely seen as unethical and is the target of widespread public disapproval. There is at present no definitive ethical verdict on the most prevalent form of DI, namely use of drugs in pursuit of enhanced cognitive performance (e.g., Farah, 2012; Caviola et al., 2014; Maslen et al., 2014). Our results elucidate the complex psychological processes underlying NE. It is likely that there are various forms of NE; regardless of whether one analyzes behavior according to the type of drug or drug option involved or behavior according to the goal pursued. When dealing with somebody who abuses Ritalin it is important to know whether the aim is deficit recovery or mitigation (i.e., to cope with and recover from academic demands) or enhanced performance (in this example supra-normal concentration). Unregard of the pursued purpose abusing this drug is a problem. But the arguments needed to convince a person to refrain from this abuse might be different.

In conclusion the aim of this article was to propose to, first, consequently account for the motivational roots of NE behavior in future investigations. Second, we feel that the proposed approach to the research topic, namely defining NE as the non-medical use of psychoactive substances for the purpose of producing a subjective enhancement in psychological functioning and experience, will help to overcome the conceptual limitations which have hampered research dedicated to the abuse of pharmacological products for the purpose of enhancing cognitive performance thus far (Zohny, 2015). Last but not least, we have provided empirical evidence that university students using NE might be classified according to their motivation or goal, e.g., “neuroenhancers” or “fatigue-fighters” and that this captures fundamental differences in NE behavior. We believe that such forms of differentiation between users are essential to devising techniques for deterring risky behavior among university students.

## Author contributions

RB and WW developed this research question. WW conducted the empirical part of the study. RB, WW, and MZ jointly analyzed the data and cooperatively wrote this report.

### Conflict of interest statement

The authors declare that the research was conducted in the absence of any commercial or financial relationships that could be construed as a potential conflict of interest.

## Footnotes

^1^According to ICD criteria addiction (termed dependence syndrome by the WHO) is a “cluster of physiological, behavioral, and cognitive phenomena in which the use of substances takes on a much higher priority for a given individual than other behaviors that once had greater value. A central descriptive characteristic of the dependence syndrome is the desire to take the psychoactive drugs. There may be evidence that return to substance use after a period of abstinence leads to a more rapid reappearance of other features of the syndrome than occurs with nondependent individuals” (cited from “World Health Organization, Management of substance abuse, Dependence syndrome,” last modified 2016, http://www.who.int/substance_abuse/terminology/definition1/en/).

^2^Although there is no evidence that (all) eligible substances actually enhance neural activity, the umbrella term's prefix “neuro-“ seems to be acceptable here because of its widespread and established use in social science research.

^3^We compared this two-class solution with a 1-class and a 3-class solution on the basis of a sample-size adjusted BIC. The lowest value occurred for the 2-class solution (11453.57), both other solutions were close together (1-class: 13141.95; 3-class: 13223.84). The 3-class solution had *p* -values of 0.23 for both Lo-Mendel tests. Taken together these tests support the validity of the 2-class solution.
